# A comparison of four dementia palliative care services using the RE-AIM framework

**DOI:** 10.1186/s12877-023-04343-w

**Published:** 2023-10-19

**Authors:** Siobhan Fox, Jonathan Drennan, Suzanne Guerin, W. George Kernohan, Aileen Murphy, Niamh O’Connor, Aphie Rukundo, Suzanne Timmons

**Affiliations:** 1 Centre for Gerontology and Rehabilitation, School of Medicine, University College Cork, Cork, Ireland; 2 School of Nursing and Midwifery, University College Cork, Cork, Ireland; 3 School of Psychology, University College Dublin, Dublin, Ireland; 4 Institute of Nursing and Health Research, County Antrim, Ulster University, Shore Road, Newtownabbey, Northern Ireland; 5 Department of Economics, University College Cork, Cork, Ireland

**Keywords:** Dementia, Palliative Care, Community Care, Community Services

## Abstract

**Background:**

Living with a life-limiting illness, people with dementia benefit from palliative care which considers the holistic needs of the person and their family. However, little is known about how palliative care may be best provided to people living with dementia at home in the community. We examined four exemplary dementia palliative care services for people with dementia in the community, to see what activities they were providing, what were the commonalities and differences, and what lessons could be learned.

**Methods:**

A long-list of dementia palliative care services in Ireland, Northern Ireland, England, Scotland, and Wales, was identified through a survey, and four exemplar services were chosen based on criteria including: in operation >six months; provides identifiable activities; availability of routinely collected service data; not exclusively for people with dementia in final hours or days of life. Mixed-methods of data collection included interviews, focus-groups and surveys with service staff, surveys of service users, and routinely collected service data. The RE-AIM framework was used to describe and understand the sample of dementia palliative care services.

**Results:**

The four services had varied organisational structures and were led by different disciplines. However, they all provided common core activities including holistic and person-centred care, early advance care planning with service user involvement, carer support, integrated healthcare services, continuity of care, 24/7 support, bereavement support. All had needs-based referral criteria, accepting any age or dementia sub-type. All supported people with dementia to remain living at home and to have a comfortable, dignified death in their preferred place.

**Conclusions:**

An effective dementia palliative care service may take different forms. Whether the service is dementia-led or Specialist Palliative Care-led, efficacy is associated with providing a range of key activities and implementing them effectively. The data collected strongly suggests the benefits of the dementia palliative care services to a person with dementia and their families and offers valuable insight into the key factors for the establishment and successful running of such services.

## Background

Dementia is the seventh most common cause of death worldwide [1] and is one of the main drivers of the increasing demand for palliative care [2]. Despite many calls for people with dementia (PwD) to receive palliative care [3], PwD receive palliative care less often than people with malignant disease, even though they experience high symptom burden [4–6]. This care gap has been attributed to difficulties in prognostication; organisational policies and structures not in keeping with a palliative care approach; resource shortages; lack of training and education; and the extra time needed to provide good dementia palliative care (DPC) [7].

To address this, new services and/or the expansion of existing services to provide DPC are urgently needed. Most PwD live in the community, and most want to remain at home [8]; thus, services supporting PwD to live and die at home may be particularly valued by PwD and families. While research priorities for DPC have been put forward [9], much of the research is based in residential care and there are few high-quality trials of effectiveness [10] and no best-practice approach or model for providing community-based DPC.

A small number of community-based DPC services have been described in the literature [10]. These can be grouped into three model types.

- The first model type involved dementia services providing (generalist) palliative care for PwD [11–16]. These services were either aimed at PwD specifically, or at older adults including those with dementia. Typical service activities included healthcare workers (HCWs), often geriatricians, nurse specialists and others, facilitating advance care planning (ACP), providing person-centred care, family support, and for those nearer end-of-life, providing equipment and support around transition to/from hospital.
- The next model type included Specialist Palliative Care (SPC)-provided services for PwD [17–22]. The SPC team often had geriatric training and provided similar activities but also reported additional activities such as formal counselling and bereavement care, music therapy, spiritual support.
- The final type of service model involved integration of existing dementia and SPC services in the community [23–25]. Services described a varied combination of dementia nurse specialists, primary care providers, hospices, community or voluntary organisations working together to provide palliative care to PwD living at home in the community.
- Positive outcomes were typically reported for all service model types including family satisfaction with care, large proportions dying at in their preferred place, and lower healthcare costs.

In Ireland, a new national model for DPC is being developed, to complement an overarching model of care for dementia. To inform this, we surveyed dementia, palliative care, and DPC experts and service providers in the UK and Ireland; they identified key components of a community-based DPC model, including carer support; continuity of care; interventions to support meaningful living; care planning and ACP; information, education and training [26]. To build on this knowledge, we set out to examine in greater depth some exemplary DPC services for PwD in the community, to see what activities they were providing, what were the commonalities and differences, and what lessons could be learned.

## Methods

### Design

A cross-sectional, mixed-methods study was designed to describe and understand a sample of DPC services for PwD living in the community, using the RE-AIM framework [27].

### Identifying exemplar services

Within the aforementioned survey [26] of stakeholders with expertise in DPC, we asked respondents to identify existing exemplary services in Ireland, England, Wales, Scotland, and Northern Ireland. From 112 responses, a long-list of 55 services was identified. Further detail on each service was gathered via internet searches and/or by directly contacting the services. The multi-disciplinary project team identified a short-list of potential services based on the criteria: service in operation > 6 months; provides identifiable activities; availability of routinely collected service data, and ideally self- or externally-performed evaluation data (for example, patient experience, outcomes, cost); not exclusively for PwD in final hours or days of life; ideally, has a well described, sound theoretical framework. A final purposive sample of services (n = 4) was selected to provide maximum variation for location, governance/setting, and lead-discipline.

### Evaluation Framework

To structure our evaluation, we chose the RE-AIM framework. “RE-AIM” comprises: **R**each (the absolute number, proportion, or representativeness of potential beneficiaries of a service); **E**ffectiveness (the impact of the different interventions offered by the service); **A**doption (the number of institutions or clinical professionals that are willing to adopt the service program and use it regularly); **I**mplementation (whether the program is being offered as expected or according to a manual, a clinical guideline or a protocol); and **M**aintenance which is expressed at two levels (institutional: the degree to which the service is part of the established healthcare services, and individual: the long-term effects on service users) [27].

Previously, RE-AIM was most often applied in public health and health behaviour change research, but it is increasingly being used in more diverse content areas including clinical, community, and corporate settings [28], including the evaluation of a dementia service [29]. The framework developers have recently recommended a pragmatic use of key dimensions rather than comprehensive applications of all elements, and using qualitative methodologies to understand the “why” and “how” of how the desired effects came about [28].

### Data collection

Mixed-methods were used to collect data aligned to each RE-AIM component. The primary data came from interviews and focus-groups with service staff. Novel interview schedules were developed, with questions guided by examples from RE-AIM developers [30, 31]. Interviews were digitally recorded, with permission. Staff surveys (paper and electronic copies) were distributed to those who wanted to take part but were unavailable for interview/focus-group. Surveys for current or past users of the service were distributed via service staff, with a pre-paid, pre-addressed envelope for direct return to the researchers. Finally, anonymised routinely collected service data was requested, including demographics of service users, number of referrals, place of death, etc.

Data was collected between November 2019 and November 2020. For the first site (Service A), data was gathered face-to-face, however owing to the Covid-19 pandemic data collection for the remaining three sites was conducted remotely, i.e. interviews were conducted via videoconferencing, and surveys were conducted via an online survey platform. Multiple phone and video calls were held with service gatekeepers when collecting data remotely where possible, to boost rapport, however remote data collection may have affected service engagement with data collection activities.

### Data analysis

Qualitative data from the interviews, focus-groups, and surveys were transcribed verbatim, imported to NVivo software, and analysed using “theoretical” thematic analysis [32]. One researcher (AR) coded all data, with a second (SF) reviewing a sample to promote reflection. Quantitative data from the surveys were analysed with simple descriptive statistics. Routinely collected data was provided as simple numerical data.

## Results

Four exemplar DPC services were selected, two in England, one in the Republic of Ireland, and one in Northern Ireland. For context, an overview of the four services is provided in Table 1. The specific data types collected from each site are in Table 2. The primary data collected came from interviews and focus-groups with 29 HCWs associated with the services, currently or previously (e.g. one HCW from Service B was recently retired). Their staff roles are detailed in Table 3. Staff survey respondents included consultants (n = 1), Clinical Nurse Specialists (n = 2), and staff nurses (n = 3). Service user survey respondents were all from Service B and included the son/daughter of a PwD (n = 9) or spouse of a PwD (n = 1). Three services provided routinely collected data, the other service did not provide this but instead an independent quality report was available which included some of the relevant demographic information.

**Table 1:** Overview of the four different dementia palliative care services

**Service A**	Service A is a Specialist Palliative Care (SPC) service, operating from a hospice co-located with an acute hospital. The service is open to anyone with a life-limiting illness, including PwD with SPC needs living in its mixed urban/rural catchment. This service has had a progressive ethos of accepting patients with life-limiting illnesses other than cancer, including dementia, since at least 2006. Patients with advanced dementia are seen by the community palliative care team, or can be admitted to the hospice for end-of-life care. The service provides 24/7 support at end-of-life via an externally provided “night nurse” service. Service A is led by 2 Consultants in Palliative Medicine, who rotate settings and share on-call duties. The community specialist nurses in palliative care are all upskilled in dementia care. The total staffing at the service is equivalent of 46.5 whole time equivalent posts.
**Service B**	Service B was established in 2003 specifically with the mission of supporting people with advanced dementia to live in the community until death. It was championed and led by a Consultant Psychiatrist of Old Age who received a grant to establish a pilot service, which was later made permanent and funded by the then government. The service has an urban catchment area, serving 3 geographical zones of a large city (of total 32 zones). The service is open to people with advanced dementia in the last year of life. The person must have a carer (not necessarily live-in); support is provided so that the PwD can die at home. The service is primarily run by 2 staff. The consultant has a small percentage of protected time to support the service. An Advanced Dementia Nurse runs the day-to-day activities. As the service ran at different times through charity funding, there was variably additional Advanced Nurses available to take on additional caseload. There is also administrative support. The service runs Monday to Friday 9.00 to 17.00, with emergency phone support outside of this time provided by the advanced dementia nurse.
**Service C**	Service C was established in 2016, under the local primary care services, to facilitate family members of PwD continuing to support the person to live at home in the community. The service comprises a team of 6 dementia nurses, who work a mix of full- and part-time hours. They serve an urban/rural catchment area (a number of cities and towns and surrounding rural areas). This is a general dementia service, with a notable palliative care ethos (i.e. holistic needs assessments, advanced care planning, good end-of-life care), and with links to the local SPC teams. The service runs Monday to Friday 9.00 to 17.00.
**Service D**	Service D is a SPC service, operating from a dedicated wing of a regional hospice. The service has an urban catchment area (a city and its surrounds) and has been running since 2016. The hospice building is designed with consideration of the needs of PwD, and hospice staff are upskilled in dementia care. It includes inpatient hospice and day hospice units. The service is open to any PwD at any stage of illness via a hospice-based outpatient service (clinic) which is led by a Clinical Nurse Specialist in palliative care. PwD with SPC needs are also supported by a domiciliary service by a team of community Clinical Nurse Specialists in palliative care. The PwD can attend the day hospice or be admitted to the in-patient hospice for end-of-life care. Throughout the preceding year, the service had on average 7 staff running the day service for people with dementia, and approximately 250 staff associated with the running of the hospice and all its related activities.

**Table 2:** Summary of Data Collected Across all Services

	Service A	Service B	Service C	Service D
Staff interviews	8	6	6	4
Staff participating in focus-groups	5	0	0	0
Staff surveys	4	1	1	0
Service user surveys	0	10	0	0
Routinely collected data	Yes	Yes	Yes	No
Independent quality report	No	No	No	Yes

*Note: data was collected from Service A before COVID restrictions were imposed*

**Table 3:** Interview Participants’ characteristics, summarised across all four sites

	*Palliative Care related disciplines (N)*	*Dementia related disciplines (N)*	*Sub-total*
Clinical Nurse Specialist / Manager	9	4	**13**
Consultant Physician	2	3	**5**
Admiral Nurse	0	4	**4**
Social Worker	2	0	**2**
Specialist Trainee	1	0	**1**
Staff Nurse	1	0	**1**
Macmillan GP Facilitator	1	0	**1**
Healthcare Assistant	1	0	**1**
Dementia Navigator	0	1	**1**
**Sub-total**	**17**	**12**	
**Total**	**29**		

The findings are presented under each of the RE-AIM headings, in the order of Reach, Adoption, Effectiveness, Implementation, and Maintenance.

### Reach

Reach is described in terms of caseload and representativeness. Caseloads varied widely across the services. At the time of data collection, Service C, a general dementia service with a palliative care ethos, had the highest caseload with 154 active cases. At Service D, there were 60 active cases of PwD in the outpatient service at any given time. Service B, an advanced dementia home care service had an active caseload of up to 45. The SPC team at Service A had supported 33 PwD in the community, 7 PwD in hospice, and 2 who died in hospital, the previous year.

The two dementia-led services (B and C) were more flexible in their total caseloads, compared to the hospice services (A and D). The nurse running the activities at Service B described how she can take on more of the cases which require less intensive intervention:

*“we get too many referrals…[but]…you have a level of obligation, you want to help…you may have a family that the patient’s in bed, they’re settled, there’s no pain, they’re eating and drinking still…Just a telephone call once a week or once a month works really well at this point. So I’ve got quite a few of them sort of people as well as my more intense cases…Officially [my caseload] sits at about 25–30, but unofficially, sitting there on my spreadsheet we’ve got about 40–45.”* (Staff 2, Service B).

A definite dementia diagnosis was required for all services except the outpatient clinic at Service D where a status of ‘awaiting a memory clinic assessment’ sufficed. Whilst most services had referral criteria, no person was turned away completely; rather, signposting to a more suitable service usually occurred.

The services accepted referrals of PwD at different dementia stages. Services A and B typically saw people with advanced or end-of-life dementia. Services C and D reached PwD ranging from early to later stages, with the outpatient clinic in Service D working mostly with people with early-stage dementia. Three of the services (B, C, and D) accepted self-referrals.

Efforts were made to increase external cooperation. One service recognised PwD’s struggle with timely diagnosis and adapted their practice to allow General Practitioners (GPs) to formally make dementia diagnoses via the implementation of a new assessment tool. GPs adopted the tool leading to earlier diagnosis and subsequent early intervention for PwD.

All services had discharge guidelines, although these varied across services. One service used a ‘phases stage’. When a PwD stabilised, or moved back a stage, they were discharged, although with information for making direct contact with the service in the future when needed. As Service B mainly saw PwD at end-of-life, discharge rarely took place. The only reason for refusal was due to the service being full, which was an issue for the two SPC-led services (A and D).

In terms of representativeness, all four services accepted any PwD, based on their care needs, with no restrictions around age, gender, ethnicity, or dementia sub-type. Most staff across the services felt that their patients were representative of the local populations. The exception was Service C, serving a catchment of large multicultural cities and towns, where staff posited that local minority communities, such as the black and travelling communities were under-represented:

*“We have a low percentage of black British families…I don’t know whether that’s a cultural thing about accessing services generally or whether it’s a stigma thing within various cultures within that group that prefer not to acknowledge somebody’s dementia”* (Staff 22, Service C).

Staff in this service were actively engaged in outreach to improve representativeness. Across the services, other groups notably under-represented, or not represented, were PwD living alone and/or without a carer. An entry criterion for Service B, which provides intensive home care, is needing a carer, while Service C is actually a service for carers (of PwD), albeit it has many direct benefits for the PwD. People without a formal diagnosis of dementia were also posited to be under-represented.

### Adoption

Adoption was explored in terms of referrals to the services (i.e., adoption of the service by others), and adoption of certain DPC activities within and outside the service.

Many referrals for the inpatient hospice in Service A came from consultants in the acute hospital where the hospice was co-located, and more from local nursing homes and the community where there were dedicated community palliative care nurses (operating from the hospice). Services B and D accepted referrals from any HCWs that recognised the need for a referral; in Service D, many referrals to the hospice outpatient clinic came from (memory service) consultants at the time of diagnosis.

Several staff reported that the co-location of a service in a wider hospital or hospice hub increased appropriate referrals. This was largely attributed to awareness and understanding of the service’s availability and capability, and the ease of communication about referred cases with hospital staff.

While most were operating satisfactorily, no service described full adoption in terms of all referrers referring appropriately. The most common reason cited for non-referral was poor awareness, among the public and staff, regarding service availability. Staff across the services noted that, for a decreasing minority of people, words like *hospice* and *palliative care* carried negative connotation or were viewed only as relating to end-of-life care or cancer. Thus, even with service awareness, stigma could act as a deterrent.

*“there is a stigma around palliative care…and then also because [the] understanding is around it being [for] cancer, so they may think why would somebody with dementia…be referred.”* (Staff 17, Service A).

GPs were infrequent referrers to all services except Service C, who acquired their largest number of referrals from GPs (reflecting the context of that service as part of primary care). This was often attributed to a poor understanding of services’ activities, and poor GP and district nurse understanding that dementia is an incurable illness requiring palliative care even in early stages.

*“I have had some carers…say that the GP has quite blatantly refused to refer, and it’s taken me then to phone that GP and explain what we do and that it’s not in any way taking away from what they do, or from the day centre places that the patient goes too, this is an added extra on top which is only going to be a benefit.”* (Staff 28, Service D).

A senior staff member at Service B noted that some district nurses are uncomfortable referring their PwD at end-of-life to the service as it is run by mental health and not palliative care.

*“we’re under mental health, which really is just random, it is just that [the person] who started it all was…a psychiatrist but…the district nurses feel more reassured when the person is referred to the hospice, being physical health care.”* (Staff 5, Service B).

All services indicated engaging in promotional activities to increase service awareness amongst the public and HCWs. One service employed a range of strategies including posters, radio and newspaper adverts. Another reached out to different HCWs such as GPs, occupational therapists, ambulance crews, etc., to inform them about the service and its value. Participants perceived that these activities increased referrals and recommended long-term promotional strategies as part of service development.

Other factors increasing external adoption rates included relevant services being under the same governance. For example, GPs in one service area had very high adoption rates because they were founding members of the service thus had high “buy-in” to service activities. Similarly, staff outside the service were more likely to engage with the service activities if they personally knew staff within the service or had previously worked together.

Within the services, adoption of activities was very high, with many examples of activities about which staff were passionate. In services without 24/7 support, some staff adapted their independent practices to accommodate this. For example, in Service B, a senior staff member had given her personal number to families to ensure patients and carers could contact them if in crisis outside of normal service hours. This dedication was acknowledged by service users, one of whom stated that the aforementioned staff member goes *“above and beyond the call of duty”.*

Dedication and passion for their roles was a regularly cited key element by staff as necessary for service running. Similarly, staff across the services willingly adapted their roles to mitigate the effects of staff shortages.

*“As a practitioner you evolve around the roles, so you start to develop coping mechanisms of what you need to do when you haven’t got it”* (Staff 2, Service B).

However, some outside services did not always adopt DPC activities. For example, some (isolated) SPC services did not recognise dementia as an incurable illness requiring SPC input.

*“If I tried to get a place in the local hospice, for a patient with dementia, there’s all sorts of questions and they are quite reluctant really, but I get so cross with them…I say ‘look if the patient had got cancer you would accept them like that’ but it’s just not perceived as the same…it’s not perceived as a terminal illness…I just despair…”* (Staff 20, Service C).

Another prominent reason for non-adoption amongst external staff included lack of time. In environments like acute hospital services, staff simply did not have sufficient time to engage with DPC activities.

Lack of communication between services was another reason for non-adoption, such as between primary GPs and out-of-hours GPs as witnessed by staff in Service A, resulting in family members often having to communicate the PwD’s end-of-life status to ambulance crews in times of crisis. The implementation of a shared data tool communicating this status was recommended.

There were also positive examples of external adoption; for example, homecare staff demonstrated their engagement with DPC activities by rearranging their allocated hours based on PwD/family preference or working beyond their paid hours to adequately meet PwD needs.

*“Staff 14: home helps go way beyond their call of duty…they get paid for a half an hour but they / Staff 13: I’m sure some of them probably there much longer / Staff 14: you can’t walk out…And it’s very hard to do, to even give somebody a shower, a bed bath in half an hour”* (Service A).

Similarly, the Supportive and Palliative Care Indicators Tool was adopted by staff within and outside Service C, to indicate when a PwD might have limited life expectancy. GPs adopted this tool and thus were able to add patients to the end-of-life registry. Other services used “This is me” forms, and e-packs, which allowed electronic data file sharing in the service’s integrated network. Putting staff incentives in place to promote the use of palliative care tools was also successful.

Finally, external adoption rates were also higher if training was undertaken, and this in turn increased adoption rates amongst their networks as seeing the importance of DPC activities led other teams to ask for training. Services engaged in outreach to increase “grassroots” adoption, for example staff at Service C participated in dementia education at the local University.

### Efficacy

No services were able to provide quantitative data on outcomes pre-post initiation of the service, or pre-post for individual patients, or for comparison groups. Service user feedback was gathered via surveys for Service B (other services either received no survey replies, or did not have the resources to facilitate this). All service users who completed feedback on Service B (n = 10) were very satisfied (80%) or satisfied (20%) with the service overall; and 100% responded “yes” that the service benefitted their loved one with dementia and their family.

Several themes emerged from the interviews and surveys regarding PwD and carer outcomes.

***Living and dying in place of choice.*** Staff across the services indicated that most of their patients planned to remain at home when given the choice, and keeping them at home for as long as possible was considered a key outcome of an effective service. PwD were perceived to typically experience better outcomes at home amongst familiar surroundings, sounds, smells etc.

*“I had one bloke…he always liked a pint of beer and his son said when you get home we’ll have a pint of beer…so they took him home, blow me down, you go and visit him and he’s sitting in the back garden having a pint of beer. It’s pretty amazing…it was his life with his son sitting in the garden on a nice chair having a pint and then going to bed…Some people just did really, really well with that model of being at home.”* (Staff 1, Service B).

The number of PwD supported to live and die at home was high across all services, reaching 80% in Service B. Qualitative data from staff and service users supported the success of the services in end-of-life outcomes.

*“My mother was at end-of-life and me and my husband were looking after her at home. We didn’t want her to go into hospital, and this was her wish too. We were desperate and looking for someone to tell us what to do and how to do it and to reassure us that we were doing the best we possibly could. We got all of this from the service and so much more. It was incredible. It allowed us to have a wonderful, dignified passing from a non-dignifying cruel disease.”* (Daughter of PwD, Service B).

For those preferring to die at home, staff worked closely with other services such as GPs and district nurses to ensure all necessary equipment such as syringe drivers were available and ready when end-of-life was anticipated.

***End-of-life outcomes***. For inpatient units, families were encouraged to bring in people’s belongings to personalise their environment and facilitate feelings of familiarity and security, contributing to a more dignified death. Discussions regarding end-of-life care were conducted across all services, typically as part of ACP. The goal was to ensure that PwD experience the most comfortable, pain-free death in their preferred place of care. A range of positive outcomes of ACP were identified by participants, including an empowered PwD knowing that all care provided will be in line with their wishes, diminished carer guilt and distress regarding making important decisions for their loved one, less burdensome life-prolonging treatments, and an overall more positive experience for the whole family.

The effectiveness of services’ end-of-life activities was diminished without an ACP in place; a particular issue in the services targeted at end-of-life or advanced dementia as ACP needs to be done prior to their involvement. Services (e.g., A and C) were engaging actively with training and supporting GPs to undertake ACP with their dementia patients.

***Continuity of care.*** All services claimed they provided continuity of care. Services B and C were based on a key worker model, with one main point of contact and care coordinator creating a seamless pathway for families. The outcomes of this included reduced stress and burden on families, and was greatly valued by them:

*“[Our] dementia nurse regularly checks in with us to ensure all is well; [She] appears interested in our situation, demonstrates empathy, and offers guidance.”* (Service user, Service B).

Continuity of care fostered a trusting relationship between the PwD, carer, and staff, enabling better understanding of the person’s context, comparisons (before and after a new intervention) and easier discussions of difficult topics. This was especially important if the PwD was admitted to hospital. Due to unfamiliarity with the palliative model of care, external HCWs were sometimes reluctant to discharge PwD back into the community after admission. DPC service staff, who knew the PwD and their wishes personally, could advocate on their behalf to get them home for end-of-life care.

***Integrated healthcare service access.*** Across the services, effective integration of healthcare services was a key desired outcome. PwD and families benefitted from the services’ networking contacts by securing early, timely admission, or necessary equipment for returning/remaining home.

*“I just said, I think you need a hospital bed now, and his wife said it’s Friday afternoon what are you going to do, and I said well I’ll see what I can do, and I actually got one for 6 o’clock in the evening…she was amazed by that.”* (Staff 1, Service B).

In addition, two of the services provided on-call doctor services, allowing people in the community to get 24/7 support. This facility was noted as a key benefit of the services as most hospital/emergency department admissions occurred outside of the regular working hours, due to lack of service availability at this time. The out-of-hours support was also highly regarded by carers who often experienced isolation at nights.

*“I think a lot of the triggers of ambulances coming and hospital admissions, it would be of a night often or bank holiday Monday…and I would always try to say just please use our numbers first and if I need support from a GP or we do need to talk to an ambulance service you know then we’ll do so but I did really try to encourage them to use our number first.”* (Staff 6, Service B).

Other key benefits included staff advocating on families’ behalf for equipment (e.g. wheelchairs), financial grants, social care needs and overall helping with navigating the system in a way that benefits them most.

*“if you are under the hospice, so you can get night carers that you can’t get otherwise. You can get the fast track, a financial fast track to continuing care. So there are some advantages, practical advantages in referring to the hospice, so they’re helping us with that, when it’s appropriate.”* (Staff 5, Service B).

***Improved person-centred and holistic care.*** All services were considered effective in delivering person-centred, holistic care. This included providing comprehensive initial assessments to all service users. Staff credited this comprehensive approach for providing a foundation to create trusting relationships with families, directly impacting efficacy of subsequent service activities.

*“The quality of the assessment and the rapport that you can build within that couple of hours really can determine the quality and the openness from the family of accepting different interventions further down the line.”* (Staff 22, Service C).

No service employed a ‘one-size-fits-all’ approach. Service activities were guided by PwDs’ history, personality and preferences, emphasising the PwD’s independence and empowerment, resulting in effective supports. The services offered a wide range of treatments, internally and through external partnerships. As service staff got to know each patient so well, they were able to prescribe the most appropriate balance of holistic care. Hospice staff reported that PwD in their services benefitted from an array of complimentary therapies including reflexology, massage, and reiki, and that these were instrumental in reducing agitation and distress for people with advanced and/or end-of-life dementia.

*“I thought ‘let’s think outside of the box’ [the PwD] enjoys complimentary therapy, what about if we brought the complimentary therapist to the home and she delivered a complimentary therapy session 30 minutes before you arrived to get him calm, to get him settled, and then see if we are able to get the venepuncture done in an easier way…We then put it into place that if he had to have any sort of intervention that was invasive, that he was to have complimentary therapy session and the family just couldn’t believe the difference”* (Staff 28, Service D).

Some service staff felt that the most important service outcome was PwD and families feeling supported and heard, a direct outcome of providing person-centred care:

*“Stripping it back I think it’s about contact…consistency…developing that rapport and…drilling it down to basic human needs of connection and people not feeling alone…You can put all your big fancy interventions into everything you know but I think it’s just knowing that there’s somebody there for them who they can connect with and they can be open and honest with.”* (Staff 21, Service C).

***Bereavement care.*** Bereavement supports were provided by three of the services. One service offered up to three support visits from the team for the carer following a death and, if additional support was warranted, a referral to bereavement services was made. Another service offered a bereavement call and a follow-up three months later from the bereavement counselling team. Although the third service previously called everybody, due to service pressures, calls were latterly only made to those that had been involved with the service for a protracted period or where staff felt the carer was vulnerable. Carers in all services were encouraged to contact the service at any point if needed. The perceived outcomes of bereavement support including less complicated grief, and improved carers’ mental health. Bereaved carers rated this support:

*“We received bereavement support and even had an impromptu visit and follow up. We couldn’t have asked for more backup and after care.”* (Service user, Service B).

### Implementation

This was evaluated in terms of service origins and evolution, resourcing (staffing levels), ability to provide activities and meet outcomes as planned (fidelity), and adaptation.

One service was established in response to the publication of a report on high stress levels and comorbidities amongst carers for PwD in the community. Another began following feedback from carers and PwD that they had nowhere to turn for support, and staffs’ growing interest in providing home services due to observed deterioration in PwD after hospital admission. Many services had one primary person as the champion driving force, e.g. a consultant or nurse in the service.

All services evolved organically. For example, Service C was set up with a specific aim, to support families to keep PwD at home, however the development of roles of staff and activities of the service was ad hoc:

*“None of us had been Admiral Nurses before so it was kind of learning how we fitted into the landscape of things as well am and actually learning about what our role is you know what our role was to be.”* (Staff 21, Service C).

One service had cost data, although this was dated. Looking at a sample of 14 of their patients who died between 2003 and 2006, they calculated a cost saving of almost £700,000 between the cost of the home care they coordinated versus the cost of residential care.

All services were provided with no cost to users. All services were primarily state funded, with some contribution from dementia and hospice charities. Staffing varied in each service. Service B was run by a Consultant Psychiatrist working one day a week and a full-time Nurse Specialist. Service C was run by 6 dementia nurses of varying grades, 2 employed full-time, and 4 part-time. The SPC services (A and D) had greater staff numbers: they supported many more patients beyond PwD. As an example, to run the day hospice at Service D required 2 social workers, 3–4 volunteers, a complimentary therapist, a creative therapist and 2–3 nurses. Service A and D also had effective volunteer programmes to support service activities, including providing complimentary therapies or keeping PwD company while transferring from long-term care facilities or hospital. All volunteers were trained to work with PwD.

*“We’re reliant on volunteers that come in…like say people that don’t have any relatives they’d sit, chat to them, they’d do card making. Another lady who comes in [does] a lot of reflexology and massage and reiki”* (Staff 19, Service A).

All services’ teams operated as part of a wider multidimensional team with local community healthcare workers. Reflections on service implementation revealed that this multidisciplinary approach was integral to success.

The services provided a wide array of DPC activities. However, no service had a written manual describing all specified DPC activities, therefore fidelity is impossible to express numerically. By nature, as each PwD and family has different needs, services were often very flexible in the activities they provided, often employing creative solutions.

Staff interviews however provided valuable insight into factors contributing to (in)fidelity. Factors causing slippage included lack of time and resources. A lack of funding meant some services were unable to offer 24/7 phone support, impeding their goals to provide support and comfort for carers when they needed it most. Insufficient home care and respite hours made it difficult for families to keep their loved ones at home, especially at end-of-life as their needs increase. The increasingly busy caseload in one service meant that staff could no longer provide bereavement supports to everyone experiencing death.

*“We used to call to see everybody at one time after bereavement but just we’ve just got busier. We just don’t have the time anymore.*” (Participant 22, Service A).

Team dynamics across services were credited for their successful running, and teams with lower staff turnover tended to fare best. Staff reported that shared communication across different services and disciplines was integral to the smooth running of service activities, via signposting, inter-referrals and sometimes joint visits. One service implemented an integrated shared dataset system across community teams.

To allow for better service running and organisation, services made adaptations to certain staff roles. One example was the development of the role of a designated ACP staff person to reduce any uncertainty regarding whose role it is to conduct ACP discussions, which can result in them not happening. Another was a combined district nurse and Admiral Nurse role to prevent what had been experienced as a disjointed service according to staff. As another example, most services employed a dyadic approach from inception, supporting both the PwD and their family carer. Service D was initially planning to only provide a service to PwD in the outpatient service. However, the need to support carers was quickly realised, leading to the development of a similar dual approach and the service was adapted accordingly. This ability to self-assess and recognise when an element of the service could be better provided was another key to the success of services.

Staff within the services engaged with other teams to resolve missed opportunities that led to poor implementation. Services also ran internal audits to inform quality improvement.

*“We do run after-death audits which enable us to look at…what went well what didn’t go so well…how many patients with dementia…had the sort of death that they had wanted, and that their priorities were met, you know did they die in their own home, were they cared for in their own home, did they have any hospital admissions int he last two weeks of life*.” (Staff 25, Service C).

Recognising the difficult nature of these roles, most services provided staff with supervision and the opportunity to debrief. Regular educational sessions are also essential.

### Maintenance

It was beyond the remit of this research to assess maintenance at the individual level, i.e. long-term effects of the services on individuals. At the setting level, high maintenance rates were evident across the entire sample. All four services were initially commissioned by charity or health service organisations as time-limited projects, e.g., to see how local palliative care services could better serve PwD living in the community. All were either time-extended or integrated permanently into the wider healthcare service. Despite proving to be feasible and successful with high uptake of the offered service, one service (B) could only secure partial funding and had to reduce its geographical reach temporarily, until eventually the success of the project secured the remainder of the budget required for the full geographical area.

Intentions to maintain DPC services are further highlighted by some services expanding to larger areas. Services C and D expanded to cover additional catchment areas. Service C also expanded to include PwD in care homes in the area. Service B expanded its phone support service to include care homes.

## Discussion

The development of DPC services is an important element in supporting PwD to live well and die comfortably in their preferred place. Previous evidence suggested that it is feasible to deliver DPC in the community [10], however it remained unknown what overall service models of DPC might look like and there’s a lack of cost effectiveness data to inform funding decisions. This study examined four unique DPC services. While these had shared objectives, the context of each differed greatly. By applying the RE-AIM framework, we identified common factors to the successful running of a DPC service.

### Understanding a successful DPC service

Although services were structured differently, all appeared to achieve the goals of supporting a good quality-of-life and a comfortable, dignified death. In the dementia-led services, a key worker was central to success. They got to know each individual and family closely, and arranged and championed for an individualised and comprehensive care plan. In the SPC-led services, integrated care teams were critical to success, where the SPC team complemented existing dementia care teams with their palliative care expertise and additional resources. Evidence from general dementia care services suggests that: care coordination and case management increases the use of community-based services, reduces hospital admissions and delays nursing home admission; integrated care is associated with increased use of community-based services, and reduced hospital days; and all are associated with greater client satisfaction [33, 34].

The key components and activities of these “real-world” DPC services were consistent with those of “ideal” services reported in the literature, including holistic and person-centred care, early ACP with PwD involvement, carer support, integrated healthcare services, continuity of care, 24/7 support and bereavement support [26, 10]. The combination of all these activities contributed to services’ effectiveness. Empirically measuring continuity of care and person-centred care is complex, and of the European Association of Palliative Care white paper components of DPC, these are least well represented in empirical interventions [9]. However, the current study provided rich detail on how a DPC service provides person-centred and continuous care to the benefit of a PwD.

All four DPC services had clear referral criteria based on the level of care needs of PwD/families but had broad criteria in terms of user characteristics. However, certain groups may be under-represented in DPC services. Others have observed that minority ethnic groups may present later to dementia services owing to cultural reasons such as seeing dementia as a normal consequence of ageing, seeing caring for the PwD as a family responsibility, or they may experience shame/stigma in their community [35]. Further, a “double disadvantage” of having dementia and ethnic minority status may impede access to palliative care [36]. Thus, DPC services need to make concerted efforts to reach these especially vulnerable groups.

The organisational barriers to providing palliative care to PwD have been widely reported [36]. Use of the RE-AIM framework uncovered nuanced factors critical to service running. Driving the success of the DPC services were passionate and dedicated staff. Newly established services should identify a champion to promote adoption of service activities both within and outside of the service. Characteristics of effective champions might include being intrinsically motivated, persistent, enthusiastic, and highly effective communicators [37]. Education and training for all HCWs, i.e., those not directly engaged in DPC, is crucial to support referrals and adoption of service activities by external staff, as the DPC service cannot provide all care alone. This needs to include better education about dementia as a life-limiting illness in primary degree training, as well as outreach by DPC staff to related services to build familiarity and relationships.

Factors relating to a DPC service being maintained were evidence of its success, in terms of patient outcomes and cost saving, and a “champion” highlighting this impact to policy-makers and funders. Limited data was available to calculate the cost effectiveness of these services, but the literature would support that a service which enables informal carers to support PwD to stay living at home has a significant cost saving versus residential care, especially at end-of-life [38, 39].

A significant difference between the structure of included DPC services is that some focused exclusively on end-of-life and advanced dementia, whereas others took a general palliative approach to care from diagnosis. Effective end-of-life care is made possible by good post-diagnostic support in dementia. Thus, any DPC service needs to be aligned with a holistic, integrated and continuous post-diagnostic care pathway [40] to ensure the best quality of care and experience for people with dementia and their families from diagnosis until death.

### Commentary on the use of the RE-AIM framework

RE-AIM proved to be a useful tool in understanding and comparing DPC services. In line with the developer’s recommendations, we applied a pragmatic use of the key dimensions [28]. While we covered all five domains, some were addressed in more detail than others with variation in the level of data provided by services. Others have placed different degrees of focus across the RE-AIM domains, depending on if the study is designed to plan, implement or evaluate a program, with the Maintenance domain often not applied at all [40].

### Limitations

Some data types proved challenging to collect. It was not possible to get electronic patient records to address the RE-AIM domains (a barrier experienced by others [41]) and no baseline outcome data was available preceding the set-up of any of the services. We also have limited data from service users. Ethics approval stipulated that service user questionnaires be distributed and collected by service staff, thus the research team had limited control beyond preparing survey packs and sending reminders to staff. The burden of completing outcome measures on extremely vulnerable individuals may also have contributed to low response rates. The Covid-19 pandemic meant that most data were collected remotely, however efforts were made to build rapport with online participants, usually with two moderators, and over a high-quality videoconferencing platform. The pandemic may also have adversely affected service-user engagement with the evaluation, as it was a time of great fear and stress for the public.

## Conclusions

General dementia care and palliative care for a person with dementia overlap, and an effective DPC service may take different forms. However, whether the service is dementia-led or SPC-led, efficacy is associated with providing a range of key DPC activities and implementing them effectively. The structure of the service in terms of staff composition, etc., does not need to be prescriptive as different structures have proved effective. The data collected strongly suggests the benefits of the DPC services to PwD and their families and offers valuable insight into the key factors for the establishment and successful running of such services.

## Data Availability

The datasets generated and/or analysed during the current study are not publicly available due to their containing potentially identifying participant information but are available from the corresponding author on reasonable request.

## Abbreviations

ACP: Advance Care Planning
DPC: Dementia Palliative Care
GP: General Practitioner
HCW: Healthcare Worker
PwD: Person with Dementia
RE: AIM–Reach, Effectiveness, Adoption, Implementation, and Maintenance
SPC: Specialist Palliative Care
